# Periosteal Chondroma of the Fifth Finger

**DOI:** 10.5334/jbsr.3837

**Published:** 2025-02-28

**Authors:** Chendong He, Wei Yang

**Affiliations:** 1 Department of Radiology, Nanjing Hospital of Chinese Medicine, Nanjing 210022, Jiangsu, China; 2 Department of Radiology, Jiangsu Province Hospital of Chinese Medicine & Affiliated Hospital of Nanjing University of Chinese Medicine, Nanjing 210029, Jiangsu, China

**Keywords:** periosteal chondroma, cartilaginous tumor, hand

## Abstract

*Teaching point:* Periosteal chondroma is a rare type of chondroma characterized by a subperiosteal, calcified soft tissue mass that compresses the cortical bone and does not involve the medullary cavity.

## Case History

**Figure 1:**
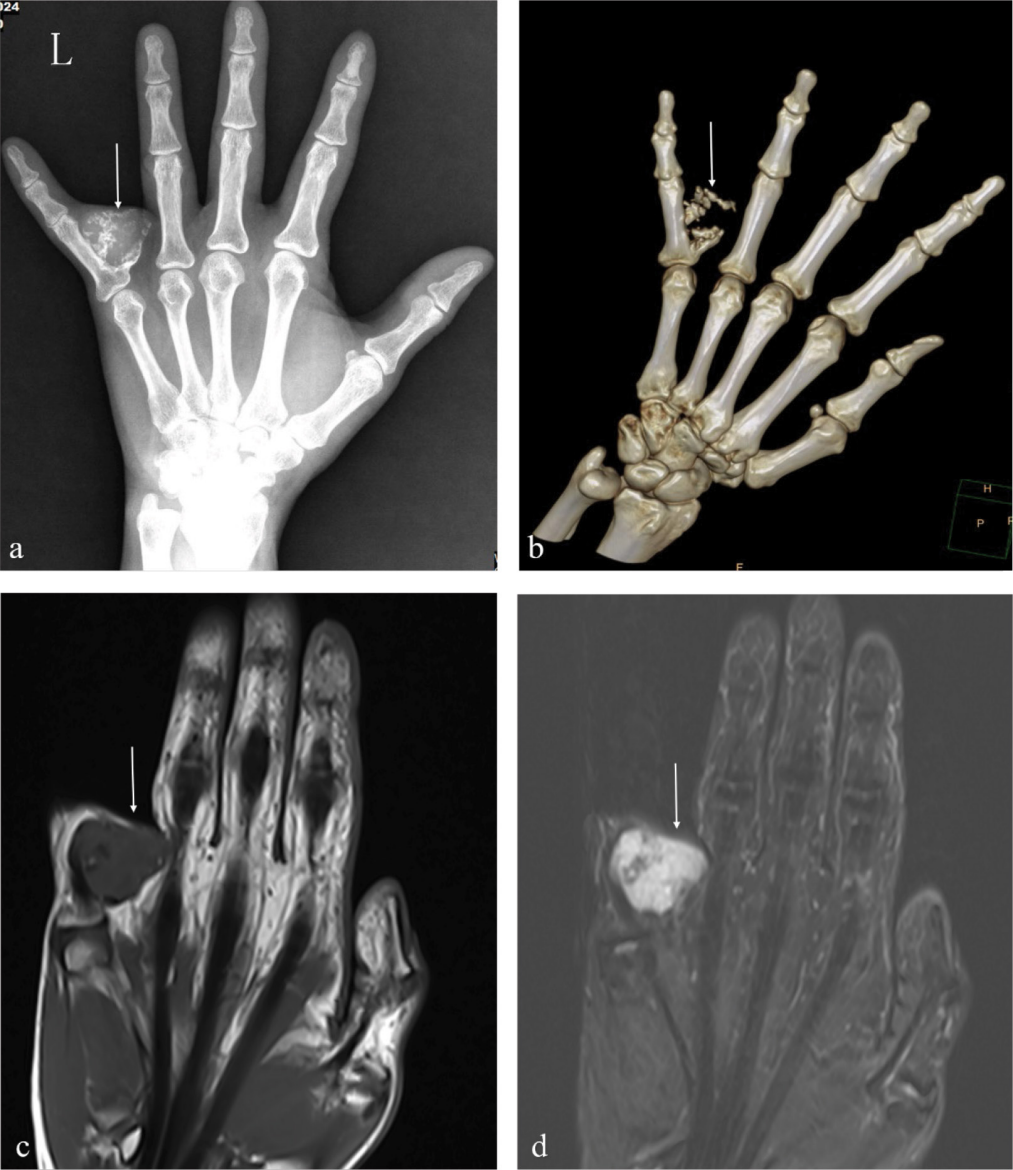
Imaging findings of the left hand. X‑rays of the left hand showed a mass at the base of the left fifth phalanx with calcification **(a)**. Volume reconstruction of left hand finger **(b)**. T1‑weight image showed a iso‑signal mass **(c)**. T2‑weight image showed a high signal mass **(d)**.

**Figure 2:**
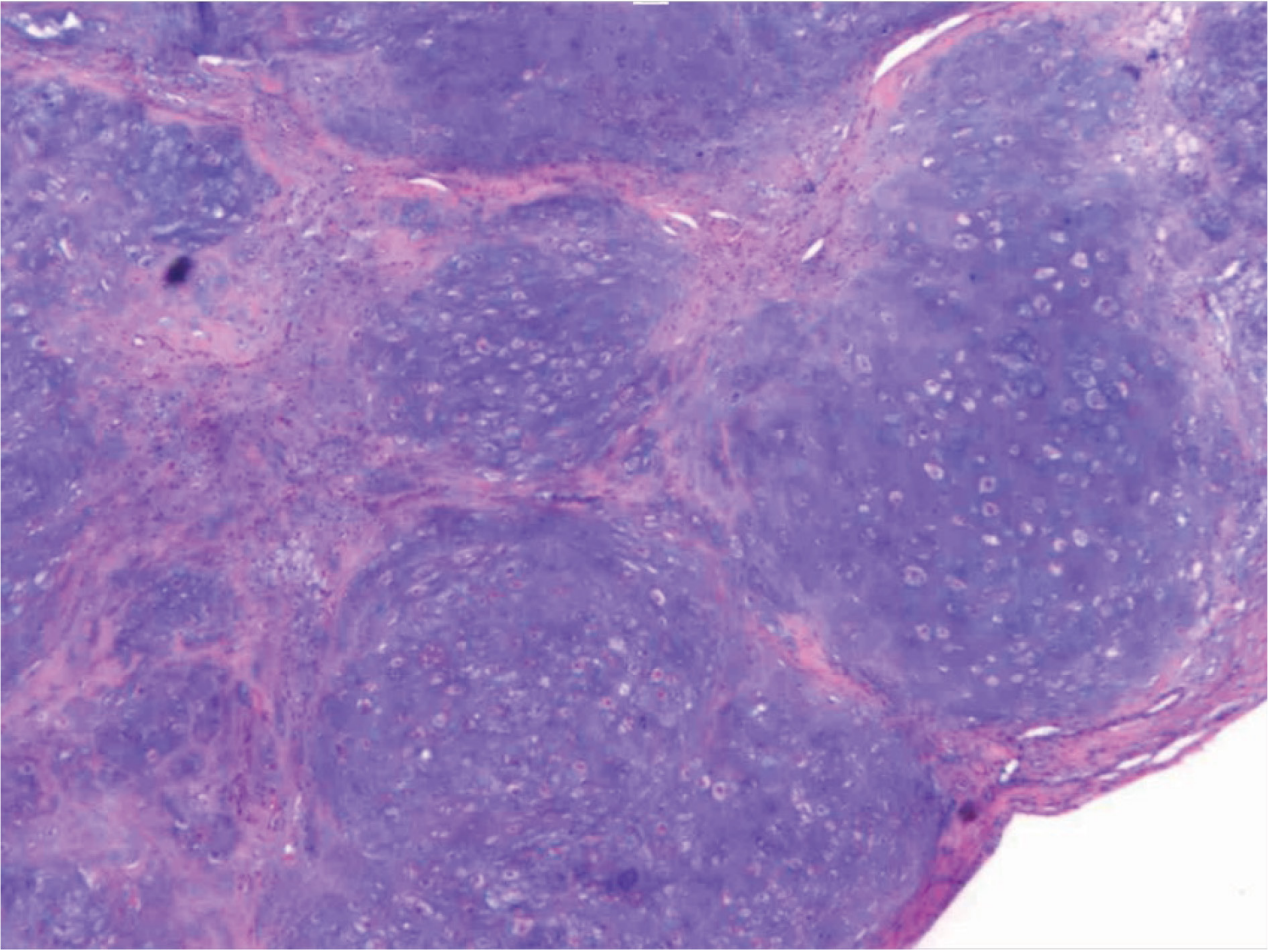
Pathological findings of the fifth finger mass. Lobules of hyaline cartilage composed of chondrocytes (x10).

A 53‑year‑old female patient presented with a lump on the left fifth finger persisting for over two years. Upon physical examination, the skin color and temperature were found to be normal, while a hard, tender mass was palpable. Notably, finger movement remained unaffected. Imaging studies, including X‑ray (Figure 1a) and computed tomography (CT) volume reconstruction of the left‑hand finger (Figure 1b), revealed a tumor adjacent to the fifth proximal phalanx of the left hand, characterized by calcified components. A magnetic resonance imaging (MRI) T1‑weighted image showed an iso‑intense mass (Figure 1c), T2‑weighted image showed a high signal mass (Figure 1d). The tumor’s base exhibited close association with the cortical bone, indicating a potential cartilaginous origin. Excision of the phalangeal lesion was performed, and pathology confirmed the diagnosis of periosteal chondroma, without evidence of atypia (Figure 2).

## Comments

Chondroma is a prevalent cartilaginous tumor with slow growth and a benign nature. Enchondroma, which arises from the medullary cavity, is frequently located in the short tubular bones of the limbs. Periosteal chondroma, a rare variant, constitutes less than 2% of all chondromas [1]. Initially referred to as juxtacortical chondroma, it originates from the cortical bone surface beneath or within the periosteum, without affecting the medullary cavity. This tumor is predominantly observed in young males, often occurring at the metaphysis or diaphysis of the proximal humerus and distal femur. There are documented instances in the ribs and acetabulum, with limited cases reported in the fingers. Typically, patients with periosteal chondroma do not exhibit significant clinical symptoms; however, in cases involving the finger joints, a painless mass may be palpable. Diagnosing this condition can be challenging, necessitating differentiation from tenosynovial giant cell tumors, Nora’s lesion, and chondrosarcoma. The three characteristic imaging features include fan‑shaped cortical bone indentation, absence of medullary cavity involvement, and a soft tissue mass containing calcified components [1]. Histologically, periosteal chondroma is composed of mature hyaline cartilage with a distinct lobulated architecture. Complete surgical excision is the preferred treatment approach, associated with a favorable prognosis.
